# Treatment Refractory Brachioradial Pruritus Treated with Topical Amitriptyline and Ketamine

**DOI:** 10.7759/cureus.5117

**Published:** 2019-07-10

**Authors:** Maja Magazin, Robert P Daze, Nicholas Okeson

**Affiliations:** 1 Medical Education, Nova Southeastern University, Dr. Kiran C. Patel College of Osteopathic Medicine, Davie, USA; 2 Dermatology, Largo Medical Center, Largo, USA; 3 Family Medicine, Largo Medical Center, Largo, USA

**Keywords:** brachioradial pruritus, pruritus, pruritus, topical amitriptyline, topical ketamine, neurocutaneous pruritus, sensory neuropathy, pruritic rash, neurocutaneous dermatoses, cervical root impingment

## Abstract

Brachioradial pruritus is an uncommon chronic neurocutaneous condition that often presents as extreme itching, burning or tingling on the dorsolateral aspect of the arm. The lack of primary skin lesions in brachioradial pruritus in addition to its poorly established pathophysiology can often lead to both diagnostic and therapeutic challenges for many physicians. Here, we present a case of brachioradial pruritus and the unique combination of topical amitriptyline and ketamine as an effective therapy, including a brief review of the literature on similar such cases.

## Introduction

Brachioradial pruritus (BRP) is a chronic sensory neuropathy that is mostly commonly seen in Caucasian middle-aged females who have a history of extensive sunlight exposure in addition to known cervical spine disease causing nerve root entrapment [1-3]. Cervical spine abnormalities such as degenerative joint disease and neural foraminal stenosis are most commonly seen at C5-C6, but the literature has also shown its association with pathology at multiple levels ranging from C3-C7 [3-6]. As cumulative lifetime exposure to UV radiation is associated with the eventual development of BRP, it should come as no surprise that symptoms commonly flare during the warmer summer months of July and August when patients are exposed to a greater magnitude of radiation in a short time frame [7]. Although medications such as topical steroids, gabapentin and pregablin are frequently used, many patients fail to respond to conventional treatment regimens. Here, a case of recurrent and refractory BRP successfully treated with a combination of topical amitriptyline and ketamine is reviewed.

## Case presentation

A 70-year-old Caucasian female with past medical history of basal cell carcinoma, degenerative disc disease at C5-C7 and autoinflammatory syndrome presented to her dermatologist in September 2017 in New England with a burning and pruritic rash of two months' duration on her right upper arm. She was diagnosed with BRP and treatment was attempted with multiple topical steroids, chiropractic manipulation, capsaicin, a menthol-based spray, NSAIDs, and gabapentin over a period of several months with no relief. Her symptoms eventually spontaneously subsided in January 2018, but then they returned once again in July 2018, at which point she came to the clinic and asked for alternative solutions. The patient otherwise felt well and denied any fevers, chills, fatigue, night sweats, unexplained weight loss or shortness of breath. Social history was significant for extensive solar UV radiation exposure in her younger years and moderate alcohol use. Medication at the time of presentation included low-dose gabapentin 200 mg and cyclobenzaprine 10 mg at bedtime for her chronic neck pain.

**Figure 1:**
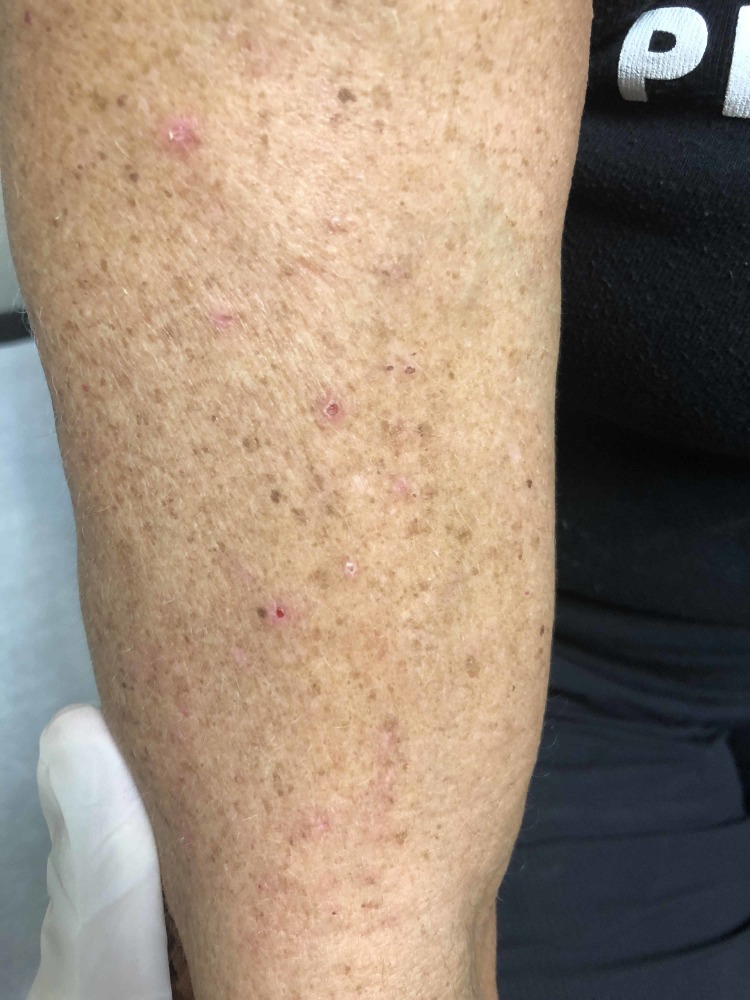
Scattered excoriations on a faint erythematous background on the right upper dorsolateral arm along the C5 and C6 dermatome prior to the initiation of treatment.

**Figure 2:**
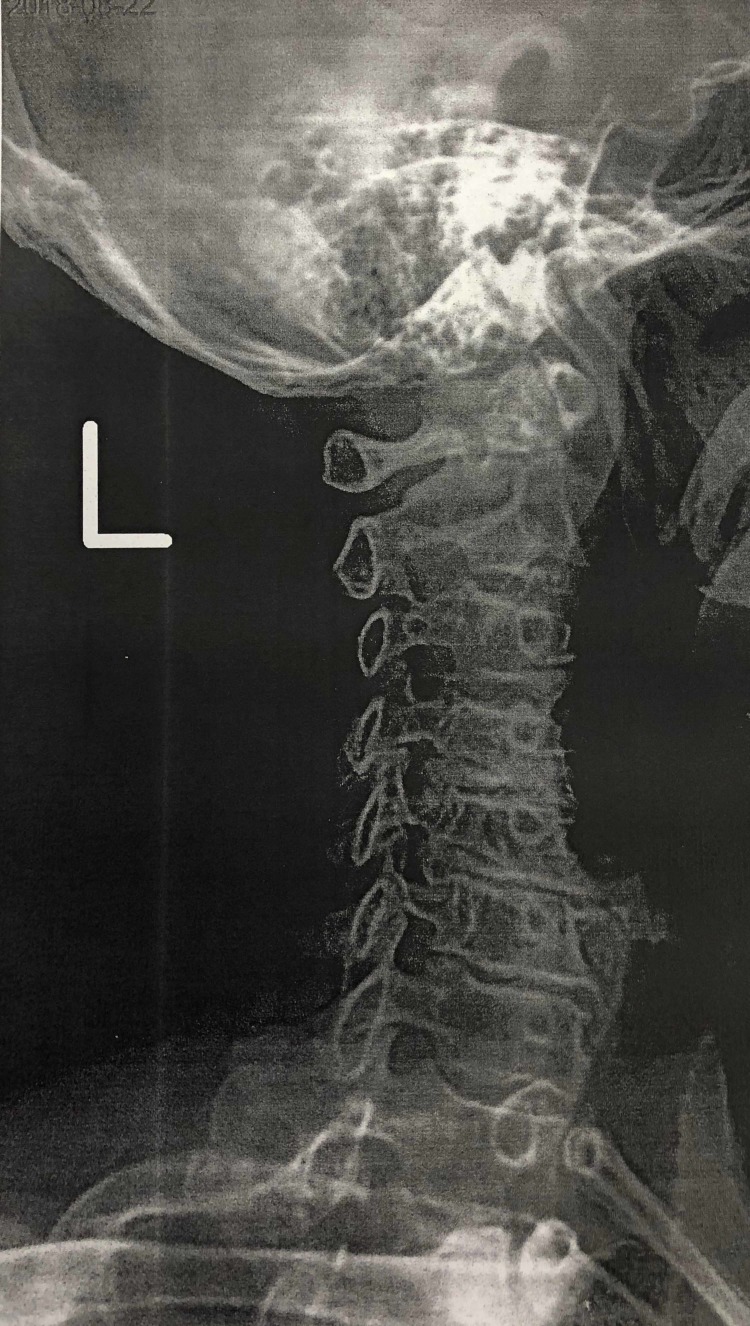
Left cervical spine x-ray demonstrating moderate to severe degenerative changes at the level of C4-C7 with intervertebral foramen narrowing, consistent with symptomatic cervical spine disease.

The patient’s presentation supported a clinical diagnosis of recurrent brachioradial pruritus. Skin examination showed scattered excoriations on a faint erythematous background on the right upper dorsolateral arm along the C5 and C6 dermatome, which were consistent with secondary cutaneous changes with no evidence of primary lesions (Figure 1). There was tenderness to palpation along C2, C3, C5, and C6 with bilateral C-spine paraspinal tightness. Radiographic imaging revealed moderate to severe degenerative changes at the level of C4-C7 with bilateral intervertebral foramen narrowing, which was worse on the left (Figures 2). The lack of primary dermatitis in conjunction with her history of symptomatic cervical spine disease and extensive sunlight exposure once again favored the diagnosis of BRP.

**Figure 3:**
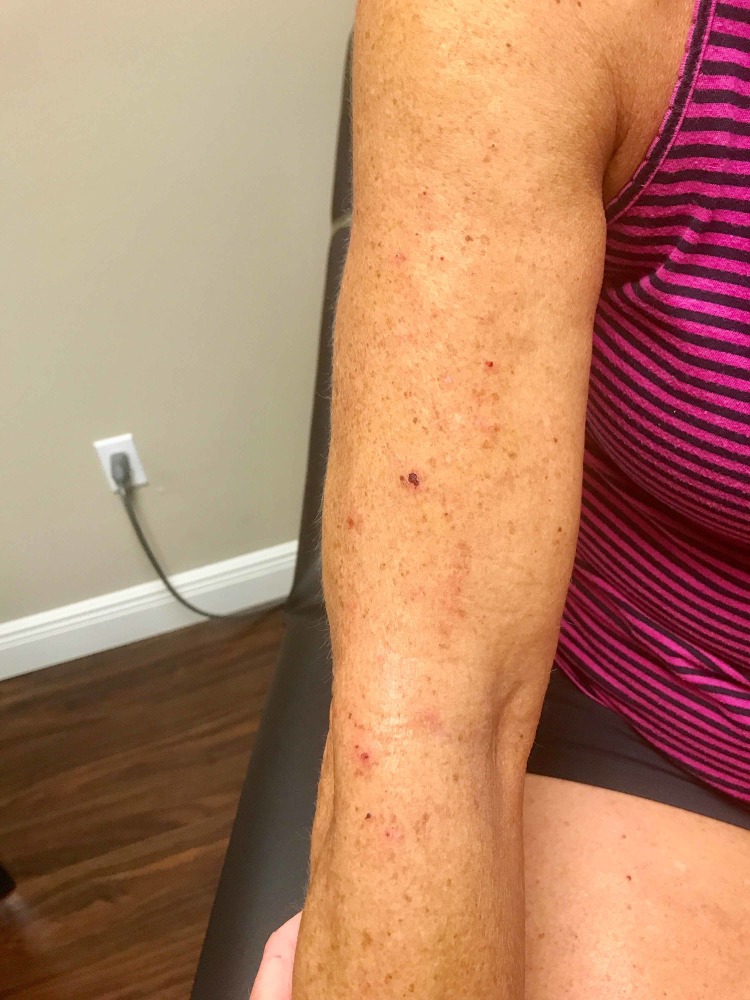
One-week follow-up demonstrating mild improvement of excoriations.

**Figure 4:**
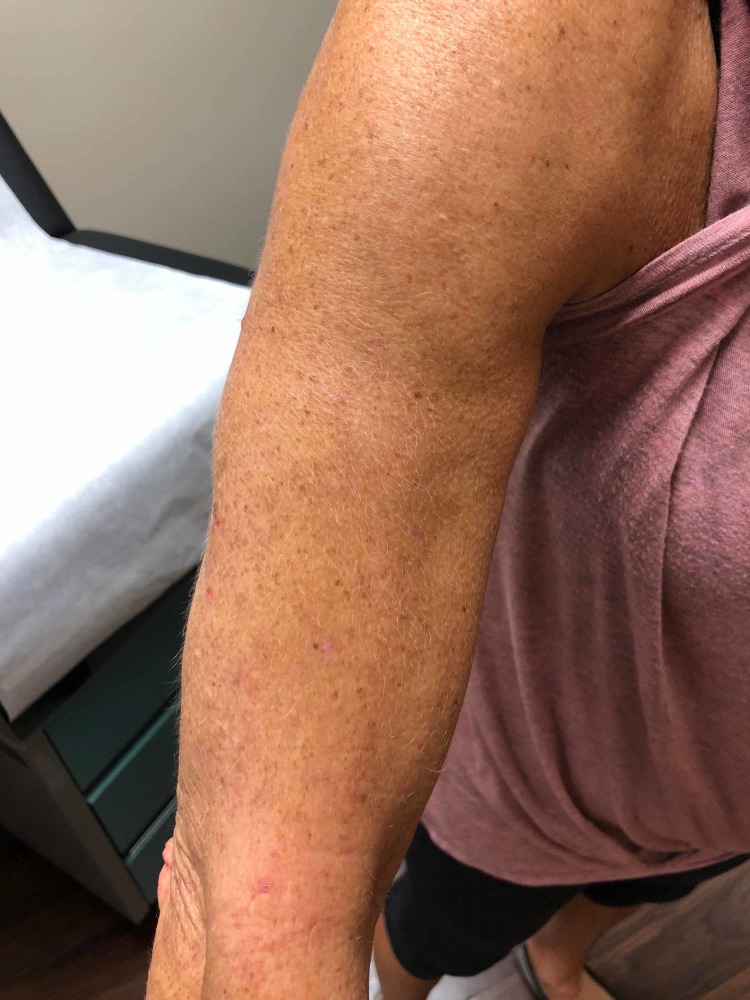
Five-week follow-up showing significant improvement of previous excoriations with no new lesions.

Due to the prior failures of several therapies, she was started on a unique topical combination of amitriptyline 1% and ketamine 0.5%. The patient was advised to apply this treatment to her arms 3-4 times daily for symptomatic relief. The patient reported immediate improvement of pruritus on Day 1 from a baseline level of 6/10 to 2/10. A one-week office follow-up showed mild improvement of the excoriations (Figure 3). Furthermore, the patient reported that she did not experience any symptoms of pruritus, such as burning or tingling during the entire fourth week of treatment. The patient also experienced a period of several consecutive days during week 5 where she was asymptomatic as well. Examination during the five-week office follow-up showed resolution of the excoriations on her arm (Figure 4). The patient noted that she often found her symptoms worsening with increased levels of stress. The only side effect reported was a tightness sensation on the area of the skin where the medication was applied.

## Discussion

This case presents a rare chronic sensory neuropathy which is poorly understood. However, recent research has proposed a theory that heavy UV radiation leads to solar damaged nociceptors capable of firing spontaneously, which could lead to the pathogenesis of this disease [2,8]. Targeting these damaged nociceptors and preventing them from firing can potentially lead to symptomatic improvement. However, one of the therapeutic challenges lies in the lack of awareness of the use of certain medications in their non-traditional forms. Amitriptyline and ketamine are most commonly known for their systemic influences, but previous studies have highlighted their alternative effects in a more localized neuropathic fashion [9-10]. In its topical formulation, amitriptyline blocks voltage-gated sodium channels and would thus be capable of preventing the spontaneous depolarization of the solar damaged nociceptors in patients with BRP [9]. In its systemic use, amitriptyline is a tricyclic antidepressant commonly used in treating depression that is unresponsive to preferred therapies such as selective serotonin reuptake inhibitors (SSRIs) and selective serotonin-norepinephrine reuptake inhibitors (SNRIs). It is also used to treat other conditions inclusive of pain syndromes, enuresis, and insomnia [10]. Its activity is thought to relate to its inhibition of 5-HT and norepinephrine reuptake. The mechanism of action of topical ketamine is similar to its systemic form where it acts as an N-methyl-D-aspartate receptor antagonist preventing transmission of signals from presynaptic nerve impulses [11]. The action of these two drugs on the cutaneous nerves involved in the pathogenesis of symptomatic BRP can ultimately provide relief from the intractable pruritus and burning and tingling sensations experienced in this chronic sensory neuropathy. Another noteworthy therapeutic barrier in the management of this disease is the availability of this pharmacological combination to the general population and the financial capabilities of the patient. Topical amitriptyline and ketamine are only available in private compounding pharmacies which often do not accept insurance as a form of reimbursement.

During the literature review, one other study was found in which a patient with BRP was successfully managed with topical amitriptyline 1% and ketamine 0.5% [5]. The authors of this study highlighted another case report which used the same dosage to treat refractory erythromelalgia pain, a neuropathic condition also thought to be influenced by irritated nocioceptors [12]. In the treatment of post-herpetic neuralgia, one study demonstrated the use of a higher dose of amitriptyline 4% and ketamine 2% to be effective [13]. Furthermore, a double-blind, randomized, placebo-controlled study examined the effect of topical formulations of 2% amitriptyline, 1% ketamine, and a combination of amitriptyline 2% and ketamine 1% on pain reduction in neuropathic conditions. The three-week study ultimately found no significant differences in pain reduction among the three treatment groups [14]. A subsequent study demonstrated that topical amitriptyline 2% and ketamine 1% was associated with significant long-term improvement of neuropathic pain when given over a period of 6-12 months with no evidence of significant systemic absorption [14]. In this study, a concentration of amitriptyline 1% and ketamine 0.5% was chosen based on a combination of these previous studies. However, it may be possible to use stronger concentrations of amitriptyline and ketamine while treating a variety of neurocutaneous conditions.

Gabapentin is an anticonvulsant that has also gained popularity in recent years for its influence on neuropathic pain and various dermatological conditions associated with pruritus. Bueller et al. was the first to introduce the use of gabapentin in the successful treatment of BRP and suggested an effective dose to be 900-1800 mg daily in divided doses [15]. A later study reported two patient cases of BRP that responded to gabapentin at 300 mg, three times daily, and suggested a dose of 300 mg once daily, titrated over three days to a dose of 900 mg daily given in three divided doses [16]. The patient presented in this case report was previously unable to tolerate higher doses of gabapentin due to systemic side effects. Pregablin is another well-known antiepileptic agent similar to gabapentin that has also been shown to be effective in the management of BRP [17]. Pregablin was not a therapeutic option for the above-reported patient due to the patient’s known intolerance to gabapentin. A more recent study described the use of aprepitant, a neurokinin-1 receptor antagonist FDA approved for chemotherapy-induced nausea and vomiting in treatment of BRP [18]. Further review revealed that this option would present a significant financial challenge to the general population and would be the least cost-effective strategy.

## Conclusions

In conclusion, this case presents the use of a non-traditional form of amitriptyline and ketamine in the successful management of refractory brachioradial pruritus. Additional studies are needed to compare alternative dosing of this combination in the treatment of BRP and other neurocutaneous conditions and to establish optimal dosing guidelines.
